# Supplementary material for: UV-Cured Bio-Based Acrylated Soybean Oil Scaffold Reinforced with Bioactive Glasses
Source: Polymers (Basel). 2023 Oct 14;15(20):4089. doi: 10.3390/polym15204089 (PMC10610054; doi:10.3390/polym15204089)
Supplement: Supplementary file 1 [file polymers-15-04089-s001.zip › polymers-2635072-supplementary.pdf]

polymers MDPI

Supplementary materials

UV-Cured bio-based acrylated soybean oil scaffold reinforced with bioactive glasses

Matteo Bergoglio, Ziba Najmi, Andrea Cochis, Marta Miola, Enrica Vernè and Marco Sangermano

Supplementary Figure S1: Comparison of the metabolic activity of the only hMSCs with E0

To evaluate the cytocompatibility properties of the samples (E0, A0, C0, C10, and C30), hMCS cells were seeded directly on their surfaces and incubated for 24 and 48 h. After each time point, their viability and morphology were analyzed using resazurin metabolic assay, fluorescent Live/Dead staining, and SEM, respectively. For this study, hMSC cells seeded in the wells of the 24-multiwell plate, without any samples, were used as control specimens. Their metabolic activity was measured by the intensity of the resorufin at an emission wavelength of 590 nm, which was defined as Relative fluorescent unit (RFU) value; RFU value correlates to the number of viable cells that can metabolize resazurin to the fluorescent components resurofin (as explained in detail in the manuscript). Since the RFU values of the only hMSCs (without any samples) are statistically similar to the ones of the cells attached to the surfaces of E0s ($p>0.05$, Supplementary Figure 1), E0 samples were considered control samples in *In vitro* evaluation of cytocompatibility properties.

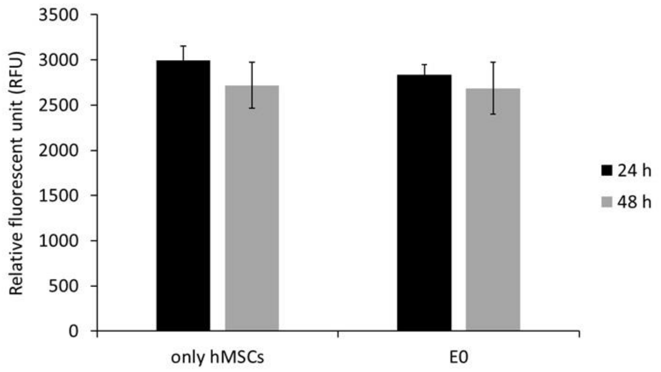

Supplementary Figure S1. Comparison of metabolic activity of the only hMSC cells (without any samples) with the one of the cells attached to the E0 samples after 24 and 48 h of incubation.

Supplementary Figure S2: Fluorescent Live/Dead assay

As mentioned above, after 48 h of incubation, the viability of the cells attached to the surfaces of the samples was visually checked using fluorescent Live/Dead staining and the results are presented in Supplementary Figure 2. These results confirmed the results obtained from the metabolic activity and SEM demonstrating the high number of viable cells (colored green) attached to the surfaces of the C30 in comparison with the other samples. Since no statistical difference in the metabolic activity between only hMSC cells and the cells attached to the E0 surface was observed, E0 samples were considered control samples.

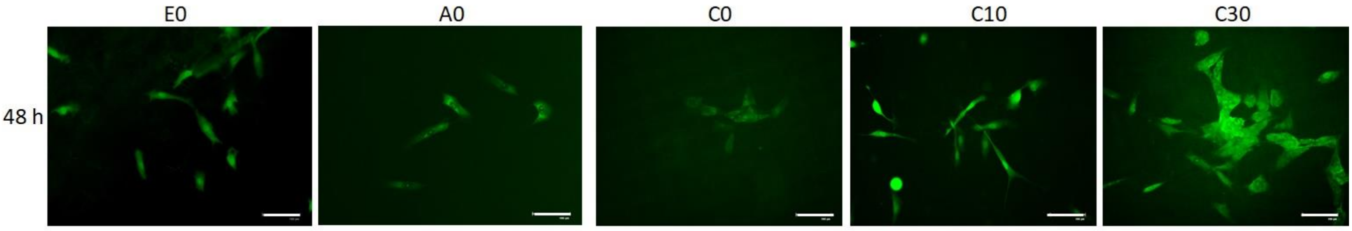

Supplementary Figure S2. Fluorescent Live/Dead assay of the cells attached to the surfaces of the samples after 48 h of incubation; Scale bar = 100 μm.
